# Current Trends of Enterococci in Dairy Products: A Comprehensive Review of Their Multiple Roles

**DOI:** 10.3390/foods10040821

**Published:** 2021-04-10

**Authors:** Maria de Lurdes Enes Dapkevicius, Bruna Sgardioli, Sandra P. A. Câmara, Patrícia Poeta, Francisco Xavier Malcata

**Affiliations:** 1 Faculty of Agricultural and Environmental Sciences, University of the Azores, 9700-042 Angra do Heroísmo, Portugal; brunasgardioli@gmail.com (B.S.); sandra.pa.camara@uac.pt (S.P.A.C.); 2 Institute of Agricultural and Environmental Research and Technology (IITAA), University of the Azores, 9700-042 Angra do Heroísmo, Portugal; 3 Microbiology and Antibiotic Resistance Team (MicroART), Department of Veterinary Sciences, University of Trás-os-Montes and Alto Douro (UTAD), 5001-801 Vila Real, Portugal; ppoeta@utad.pt; 4 Associated Laboratory for Green Chemistry (LAQV-REQUIMTE), University NOVA of Lisboa, 2829-516 Lisboa, Portugal; 5 LEPABE—Laboratory for Process Engineering, Environment, Biotechnology and Energy, Faculty of Engineering, University of Porto, 420-465 Porto, Portugal; 6 FEUP—Faculty of Engineering, University of Porto, 4200-465 Porto, Portugal

**Keywords:** dairy *Enterococcus*, starter culture, adjunct culture, protective culture, probiotics, opportunistic pathogen, pathogenicity determinants, antimicrobial resistance

## Abstract

As a genus that has evolved for resistance against adverse environmental factors and that readily exchanges genetic elements, enterococci are well adapted to the cheese environment and may reach high numbers in artisanal cheeses. Their metabolites impact cheese flavor, texture, and rheological properties, thus contributing to the development of its typical sensorial properties. Due to their antimicrobial activity, enterococci modulate the cheese microbiota, stimulate autolysis of other lactic acid bacteria (LAB), control pathogens and deterioration microorganisms, and may offer beneficial effects to the health of their hosts. They could in principle be employed as adjunct/protective/probiotic cultures; however, due to their propensity to acquire genetic determinants of virulence and antibiotic resistance, together with the opportunistic character of some of its members, this genus does not possess Qualified Presumption of Safety (QPS) status. It is, however, noteworthy that some putative virulence factors described in foodborne enterococci may simply reflect adaptation to the food environment and to the human host as commensal. Further research is needed to help distinguish friend from foe among enterococci, eventually enabling exploitation of the beneficial aspects of specific cheese-associated strains. This review aims at discussing both beneficial and deleterious roles played by enterococci in artisanal cheeses, while highlighting the need for further research on such a remarkably hardy genus.

## 1. Introduction

Enterococci are part of the subdominant microbiota of many artisanal cheeses [1], where they play an important role in the development of sensorial properties [1], help modulating the cheese microbiota [2] and may play a role in controlling pathogenic as well as deterioration microorganisms [3]. Their evolutionary history has primed them to resist (harsh conditions), adapt (to stress), and persist (in difficult environments). It has endowed this genus of commensal gut bacteria with plastic genomes and a notable capacity to trade genes by horizontal transfer events, both of a homologous and heterologous nature [4]. The adaptation to their hosts provided them the opportunity to become important opportunistic pathogens [4]; the ability to easily acquire genes by horizontal transfer allowed them to build up a considerable resistome [5], as well as several putative determinants of virulence [6]. On the other hand, enterococcal strains have been used as adjunct cultures, probiotics and may have beneficial effects upon several aspects of their human hosts’ health [7]. The impact of the enterococcal microbiota of artisanal cheeses on the health of their hosts is still the object of debate, despite the wealth of knowledge gathered in recent years on their presence, technological properties, potential health benefits, antibiotic resistance, and carriage of virulence factors [7]. Due to the uncertainty on their safety, enterococci do not possess QPS status in the EU and are not Generally Regarded as Safe (GRAS) in the USA. The lack of a recognized safety status as a genus has hampered their use as industrial food cultures, despite their potential benefits [8]. A better perception of their roles in the cheese system is required to both eventually allow for their industrial use and to understand their potential role as reservoirs of genetic determinants of antibiotic resistance and virulence. This review focuses on enterococci in artisanal cheeses, with the aim of thinking over their role(s) in this setting and exploring their current potentialities.

## 2. The Genus *Enterococcus*—A Bacterial Group That Has Evolved for Resistance

As a genus, enterococci have a relatively short history, most of which intertwined with that of other Gram-positive cocci, especially streptococci. The terms “streptococci” and “enterococci” first appeared during the Golden Age of Microbiology. “Streptococcos” has been employed for the first time in 1874, by Billroth [9], a contemporary of Louis Pasteur, to describe cocci arranged in chains that he observed in wounds. A decade later, streptococci were described as a genus, in Rosenbach’s work on bacteria from suppurated wounds [10]. The first mention to the term “enterococcus” occurred less than 20 years later, when Thiercellin & Jouhaud [11] reported isolating saprophytic, potentially pathogenic cocci from the human intestines. These new cocci were then thought to be part of the *Streptococcus* genus, and received the species epithet of *faecalis* [12]. Thirty years later, when Rebecca Lancefield published her serological classification of streptococci [13] and, subsequently, when James Sherman proposed division of streptococci into four groups [14], enterococci emerged as a separate group of “streptococcal lineages”; however, they would not be afforded the status of genus on their own until the mid-80s, when, based on molecular biology approaches, Karl Schleifer and Renate Kilpper-Bälz [15] proposed that the Gram-positive cocci belonging to Lancefield’s group D constitute, in fact, a genus, and named it after Thiercellin & Jouhaud’s cocci. At that time, only two species—*Enterococcus faecalis* and *Enterococcus faecium*—were assigned to the newly proposed genus. Presently, at the doorstep of the third decade of the new millennium, it encompasses close to 60 validly published species with correct name [16].

The association between enterococci and the human intestinal habitat has been established since the early works of Thiercellin & Jouhaud [11]. More recently, research by Lebreton et al. [4,17] has shown that enterococci are indeed a native enteric genus that has colonized from there a variety of human- and non-human-related habitats. Their ability to colonize these very diversified ecological niches stemmed from their evolutionary history, which has been hypothesized to have started 500 ± 130.5 Myr, driven by terrestrialization of the animal hosts that sheltered the enterococcal ancestors [17]. Based on 16S rRNA data, enterococci seem to have arisen from *Vagococcus*-like ancestors, which in turn stemmed out of the Carnobacteriacea branch of the bacterial tree of life. *Carnobacteria* and *Vagococcus* remained associated with marine hosts [17], whereas most enterococci relate to the gut of terrestrial animals (mammals, birds, reptiles, and insects) [17,18,19]. As a result of their evolution towards hardiness, members of this genus have been found in soils [20,21], waters [22], plants [21,23], hospital environments [24], foods [8,25] and feeds [26,27], further to their association with human and animal microbiota. Enterococci do not sporulate; hence, their persistence in these seemingly hostile environments stems, in part, from their ability to enter a viable, non-culturable state [28], their notable resistance to starvation, and their capacity to withstand desiccation [17]. The plasticity of the enterococcal genome granted them not only the ability to colonize a wide range of niches, but also capacitated them to play a wide range of roles in these environments—as well as in their human hosts—where they range from harmless commensals, through opportunistic pathogens, to probiotic bacteria [29].

## 3. Raw Milk as a Source of Enterococci for Dairy Products 

Lactic acid bacteria are part of the core, mesophilic/psychrotrophic microbiota of raw bovine milk [30,31]. Although lactococci, lactobacilli, and streptococci may reach populations in the order of 10^4^ CFU mL^−1^ in raw milk, the numbers of leuconostocs and enterococci reach only 10^3^ CFU mL^−1^ [30]. 

Enterococci may be present to large numbers in dairy products (up to 10^8^ CFU g^−1^) [32]. They are among the most common lactic acid bacteria in raw milk [24], which they access from dairy environment, animals, and humans [32,33,34,35]. Despite their relation to the intestinal microbiota of humans and dairy animals, fecal contamination does not seem to play an important role upon entrance of enterococci into the dairy production chain. The milking equipment has instead been regarded as their major source in raw milk [36]. The work by Gelsomino et al. [34] has demonstrated that the milking machine and the bulk tank are important sources of enterococci for milk and dairy products. The milking environment was found to be the source of vancomycin-resistant enterococci (VRE), rather than the animals [37]. Mastitis can also be a source of multidrug-resistant enterococci [38]. The season of the year may also play a role in modulating the enterococcal diversity in raw milk [39].

Raw milk may, therefore, serve as a source of enterococci for dairy products, even when pasteurization is applied during processing a posteriori. There are indeed reports of enterococci surviving pasteurization temperatures [40]. Their thermal resistance, however, is highly variable and species-dependent, with *z* values ranging from 5.0 (*E. faecalis*) to 9.8 °C (*E. hirae*) [41]. The thermal resistance of enterococci depends also on the growth phase and previous thermal history of the cells [42]; hence, the enterococcal microbiota in pasteurized milk differs from that of raw milk. Although *Enterococcus durans* tends to predominate in the former, the most prevalent enterococcal species in the latter is *E. faecalis* [39]. Besides their thermoduric character, post-treatment recontamination also explains the presence of enterococci in pasteurized milk products, such as cheese [32], with biofilms on milk-contact surfaces acting as a potential source of high numbers of bacteria [43,44].

**Figure 1:**
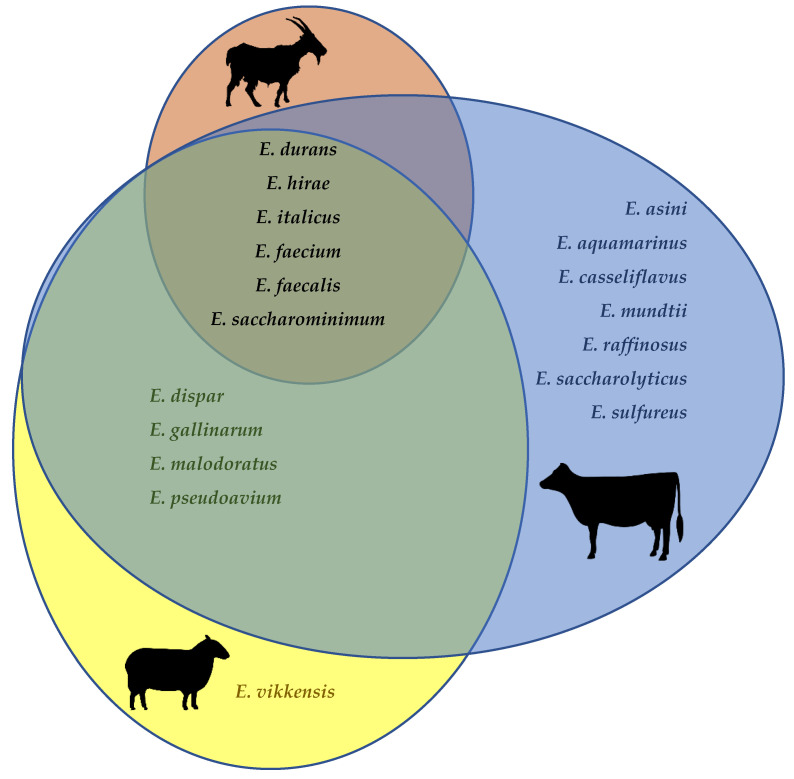
Enterococcal species described to date in raw cow’s, goat’s, and ewe’s milk [31,35,38,39,41,45,46,47,48,49,50,51,52,53,54,55,56,57,58,59,60,61,62,63,64].

*Enterococcus faecalis*, *E. faecium*, *E. durans*, *E. hirae*, as well as (albeit infrequently) *E. saccharominimum* and *E. italicus*, have been isolated from the raw milk of cows, goats, and sheep (Figure 1) [31,36,38,39,41,45,46,47,48,49,50,51,52,53,54,55,56,57,58,59,60,61,62,63,64]. Additionally, *E. dispar, E. malodoratus, E. pseudoavium* and *E. gallinarum* have been isolated both in cow’s and in ewe’s milk. *E. casseliflavus*, *E. mundtii*, *E. aquamarinus*, *E. asini*, *E. saccharolyticus*, *E. sulfureus*, and *E. raffinosus* have been described only in cow’s milk, whereas *E. vikkensis* was found just in ewe’s milk. It is worth keeping in mind, however, that the apparently lesser diversity of enterococcal species in goat’s and sheep’s milk when compared to cow’s milk may merely reflect the much fewer studies that focused on the former.

Several of the enterococcal species described in raw milk (e.g., *E. faecalis*, *E. faecium* and *E. mundtii*) are known to include strains that produce bacteriocins. Therefore, enterococci might play a role in modulating the milk microbiota, as well as the bacterial communities within dairy products, thus contributing both to their safety and to their sensorial properties [65]. Several enterococcal species from raw milk have demonstrated important technological properties with impact upon the sensorial properties of dairy products, such as diacetyl production, autolytic activity, proteolytic activity [31,66] and lipolytic activity [67], as well as probiotic potential [68,69].

## 4. The Enterococcal Microbiota of Artisanal Cheeses

Nineteen enterococcal species have been described in artisanal cheeses so far—*E. avium*, *E. casseliflavus*, *E. devriesei*, *E. durans*, *E. faecalis*, *E. faecium*, *E. gallinarum*, *E. gilvus*, *E. hirae*, *E. italicus*, *E. lactis*, *E. malodoratus*, *E. mundtii*, *E. pallens*, *E. pseudoavium*, *E. ratti*, *E. saccharominimus*, *E. sulfureus*, and *E. villorum* (Table 1) [37,73,75,76,77,78,91,92,93,95,98,99,100,101,102,107,111,116,117,118,119,120,121,122,123,124,125,126,127,128,129,130,131,132,133,134,135,136,137,138,139,140,141,142,143,144,145,146,147,148,149,150,151,152,153,154,155,156,157,158,159,160,161,162,163,164,165,166,167,168,169,170]; they correspond to ca. one third of the species recognized at present in this genus. In many cheese varieties, the most prevalent enterococcal species are *E. faecalis* and *E. faecium*—and, to a lesser extent, *E. durans* [32]. It is, however, important to note that cheaper culture-independent methodologies did not become widely available until recently, and this may have limited the number of species found so far. In the future, a growing accessibility to such methodologies may lead to reports on presence of other species, and even to discovery of novel enterococci in cheese.

## 5. The Technological Roles and Potentialities of Enterococci in Artisanal Cheeses

In many artisanal cheeses, enterococci are part of the non-starter lactic acid bacteria (NSLAB) [1] and they have, in some instances, been used as components of experimental starter cultures [32,142,171]. As part of the NSLAB, their role in development of the typical sensorial characteristics has been documented for several artisanal cheese varieties, such as Armada, Caprino, Caciocavallo Palermitano, Cebreiro, Comté, Feta, Fontina, Majorero, Manchego, Mozzarella, Monte Veronese, Pico, Serra, Venaco (Southern Europe), Ras, Domiati (Egypt), and Izmir Tulum (Turkey) [32,127,142,153,171,172]. Although their role as NSLAB in the development of flavor has been demonstrated in many artisanal cheese varieties, their potential as cheese starters is much less clear. The main roles of a starter are to promote acidification and to take part in the initial steps of proteolysis, both of which are nuclear events in maturation and essential for development of unique cheese flavor, texture, and mouthfeel [173].

Isolation of enterococcal strains with good acidification properties from artisanal cheeses is not frequent, and comprised only 3.6 to 11.6% of the enterococcal isolates in Pico and Pecorino Abruzzese cheeses, respectively [174,175]. In several studies, the enterococci have been reported to exhibit a poor acidifying capacity, with only a small percentage of the isolates being able to produce acid enough for pH to drop below 5.0–5.2 upon incubation for 16–24 h at 37 °C [176,177,178]. Some studies have shown that *E. faecalis* acidifies skim milk faster than *E. faecium* [85], but others have unfolded a wide inter-strain variation [179].

Casein degradation plays a role not only upon cheese flavor and texture, but also upon the accumulation of bioactive peptides, with potential health benefits, within the product matrix [7]. Caseinolytic strains have been isolated from artisanal cheeses to variable proportions (17–95%) of the total enterococcal isolates [153,174,175,180]. The *gelE* gene, frequently regarded as a virulence factor, is involved in caseinolysis when the enterococci grow in dairy substrates. This gene is more frequent in *E. faecalis* than in other enterococcal species, in agreement with the higher proteolytic activity described for this species [7,179,181,182]. The expression of *gelE* has been associated, in recombinant enterococcal strains, to the production of milk protein hydrolysates with high inhibitory activity against the angiotensin-converting enzyme (ACE) [183]. In addition to the effect of the species, proteolysis in enterococci seems to also depend on the strain [7].

Enterococci are auxotrophic for several amino acids [184]. Therefore, their ability to grow and reach high populations in dairy matrices requires that they possess aminopeptidases to release essential amino acids from the casein-derived peptides as outcome of primary cheese proteolysis [185]. Casein degradation by enterococci is first accomplished by the extracellular Clp proteolytic complex. The resulting oligopeptides are then brought into the cell by several transport systems (Opp, Dpp, and DptT), where they are further degraded by aminopeptidases (Pep A, Pep B, Pep C, Pep F, Pep O, Pep Q, Pep S, Pep T and Pep V); they will eventually serve as a source of nitrogen for enterococcal growth, and function as precursors for flavor compounds [186]. Moreover, free amino acids can be converted to biogenic amines by enterococcal decarboxylase systems. Amino acid decarboxylation is activated in acidic environments, and may help the enterococcal cell maintain its pH homeostasis under such conditions. It may also support primary metabolism under nutrient depletion conditions [186]. Enterococci have often been identified as one of the main biogenic amine producers in cheese; synthesis of histidine decarboxylase, tyrosine decarboxylase, lysine decarboxylase, ornithine decarboxylase, and agmatine deiminase, as well as production of the corresponding biogenic amines (histidine, tyramine, 2-phenylethylamine, cadaverine, and putrescine) have been described for several species of this genus [187]. Nevertheless, aminopeptidase activity is a desirable trait for cheese starter cultures, since said enzymes release important flavor compounds and precursors thereof (i.e., small peptides and free amino acids) [173]. Furthermore, their activity may contribute to prevent flavor defects. Aminopeptidase P, for instance, contributes to reduce bitterness in cheese, by hydrolyzing proline-rich oligopeptides derived from proteolysis of casein [188]. The aminopeptidase activities of enterococci from artisanal cheeses seem to vary in a strain-, rather than species-dependent manner [142,172,174,175].

Given the intracellular location of aminopeptidases, the autolysis of enterococcal cells is important for cheese maturation, since it promotes contact of these enzymatic systems with their substrates, and consequently assists in flavor development by accelerating peptidolysis [189]. Starter autolysis has also been reported to assist in the control of bitterness defects in cheese [190]. Autolytic activity is highly dependent on the LAB strain [190]. Strong autolytic activity has been reported in enterococci from artisanal cheeses [154,191], which has been linked to higher aminopeptidase activity in the case of cheese slurries [191].

Due to their generally low acidification capacity and limited proteolytic properties, enterococci are not often regarded as important elements of the primary starter cultures for cheesemaking [32]. They may, however, offer a promise either as adjunct [32,82,192,193] or as protective cultures [32,176]. Adjunct cultures are deliberately added to increase the intensity and help achieve a balanced cheese flavor. They are often selected among cheese NSLAB; hence, metabolic activities such as lipolysis, esterase activity, proteolysis, amino acid degradation, and citrate metabolism are of interest when screening for cheese adjunct cultures [194]. It has also been suggested that exopolysaccharide (EPS) production may be an interesting trait, especially for low-fat cheese varieties, where EPS would help improve textural properties and water holding capacity [195].

Lipid metabolism by the cheese microbiota gives a considerable contribution to the flavor and texture of many cheese varieties, via hydrolysis of the milk lipids retained in the cheese matrix and thereby releasing free fatty acids (FFA). These FFA are, in turn, metabolized to volatile compounds (methylketones and thioesters) that take part in cheese flavor. Although lipases act on emulsified milk fat, the esterases attack the dissolved lipid fractions [7]. Generally, cheese LAB (including the enterococci) are regarded as weakly lipolytic [7]; however, there are reports of strong lipolytic activity among enterococcal strains isolated from some cheeses [160,177], which may indicate that this trait is species- and strain-dependent. The enterococci have been described as possessing a higher esterolytic activity than the remaining LAB genera [7,160]; however, their activity seems to be mostly limited to short-chain fatty acids [160].

Another activity of enterococci with an impact upon cheese flavor is their citrate metabolism, which releases several C-4, volatile compounds (e.g., diacetyl, acetoin, and butanediol). Furthermore, the CO_2_ released during citrate use takes part in the formation of the “eyes” (cavities) that are typical of such cheese varieties as Gouda and Danbo [196]. The production of diacetyl and, to a lesser extent, of acetoin have been described in enterococci from cheese [142,153,175,197,198]. Additionally, annotation of enterococcal genomes has revealed genes that encode for enzymes involved in acetaldehyde, diacetyl, and acetoin [186,199].

Some LAB can accumulate extracellular heteropolysaccharides; besides their protective functions, they take part in adhesion to surfaces and in biofilm formation, thus assisting EPS-producing strains in colonizing their environments, including their hosts’ gastrointestinal tract (GIT) [200]. Due to their emulsifying, thickening, and anti-syneresis properties, these EPS can modify the texture and rheological properties of cheese [201]. The ability to produce EPS has been reported in enterococci from artisanal cheeses [202,203], and could be a positive trait for their use as adjunct cultures, especially in low-fat products [204].

Many strains of enterococci associated with cheese produce a wide diversity of bacteriocins (enterocins) bearing a broad-spectrum activity against several Gram-positive foodborne pathogens, including *Listeria monocytogenes*, *Staphylococcus aureus*, *Bacillus cereus*, clostridial endospores and vegetative cells, as well as other LAB [3,32,205]. Enterocin production may partly explain the success of enterococci in colonizing and reaching sizable numbers in cheese [3]. Enterocin diversity in enterococci is thought to stem from the remarkable ability of the bacteria in this genus to trade genetic material. For instance, the genetic determinants for enterocins EJ97 and AS-48 are encoded in conjugative, pheromone-responsive plasmids, thus making their transfer between enterococci fast and efficient [3]. As most bacteriocins, enterocins primarily target the cell membrane, where they form pores, thus leading to leakage of essential intracellular constituents while depleting transmembrane potential and/ or pH gradient [206,207]. One notable exception is enterolysin A, which attacks the cell wall [208]. In cheese, bacteriocin-producers may contribute to modulate the microbiota during ripening [2], promote the autolysis/increase cell permeability of starter and NSLAB (with the concomitant release of intracellular enzymes) [190,209,210], and inhibit pathogenic and deterioration microorganisms [58,211,212,213,214]. Enterocin production is, therefore, germane for adjunct cultures with protective functions. Several reports describe the experimental use of bacteriocinogenic *E. durans*, *E. faecalis*, *E. faecium*, and *E. mundtii* strains as cheese protective cultures [58,212,213,214,215]. However, some enterococci can act as human nosocomial, opportunistic pathogens; due to their propensity to trade genetic material, they may act as reservoirs of virulence and antibiotic-resistance genes [214,216,217]. Therefore, the genus has not been granted a Qualified Presumption of Safety (QPS) status. A thorough investigation of the safety-related phenotype and genetic determinants is indispensable for each enterococcal strain before equating its application in food. With these concerns in mind, the usage of purified or semi-purified enterocins as antimicrobial food additives has, in some instances, been recommended instead of its in situ production [217,218].

## 6. Enterococci and Potential Health Benefits of Dairy Products

Besides contributing to the sensorial properties of artisanal cheeses, the action of enterococci on the protein fraction of milk, curd and maturating cheese may lead to accumulation of bioactive peptides with potential benefits for the consumers’ health. Bioactive peptides are short protein fragments (2–20 amino acid residues) that can exhibit hormonal or drug-like effects [219,220]. Milk proteins are the most exploited source of peptides with antihypertensive, hypocholesteremic, antihyperglycemic, immunomodulatory, antimicrobial and antioxidant activity. Due to their proteolytic activity, several LAB (including enterococci) produce bioactive peptides when growing in milk [7].

The production of antihypertensive peptides by enterococci in milk fermentations has been demonstrated both in vitro [7,221,222] and in animal models [223,224]. Torres-Llanez et al. [225] reported that fresh cheese containing *E. faecium* had high in vitro inhibitory activity against the angiotensin-converting enzyme (ACE); in another study, the consumption of a traditional Norwegian cheese (Gamalost) was found to decrease blood pressure in 128 subjects with a mean age of 51 years [226]. In the review by Baptista et al. [227], the presence of ACE-inhibitory peptides was described in Valdeón, Grana Padano, Maasdam, Gorgonzola, Cheddar and Parmigiano Reggiano. These very short peptides (3–7 amino acid residues) were all derived from β-casein [227]. Cheese maturation was shown to promote release of bioactive peptides [228,229], and so did in vitro digestion, thus confirming the potential of cheese as vehicle for their administration.

Enterocin production may provide a competitive advantage within the GIT to the producing strains, owing to their antimicrobial activity. Consequently, enterococcal strains that produce this type of antimicrobial peptides are better equipped to establish themselves in the GIT of the host, to directly inhibit pathogens and to modulate the host’s gastrointestinal microbiota [230]. The potential for selected enterococci toward modulation of the GIT microbiota in the host, when administered orally, has been demonstrated in animal models [231,232].

Albano et al. [233] demonstrated that an *E. lactis* strain decreased the cholesterol concentration in broth and as part of a cheese adjunct culture, whereas oral administration of certain *E. faecalis* or *E. faecium* strains led to a significant decrease in serum cholesterol levels of hypercholesterolemic animal models [232,234]. In the case of *E. faecium*, cholesterol excretion was promoted; cholesterol degradation and transportation genes were up-regulated, while genes involved in cholesterol synthesis were down-regulated [234]. Translocation of the orally administered bacteria, which could unfold an invasive potential, was not observed and low-density cholesterol levels decreased in a mouse model [233].

The products of milk fermentation by *E. faecalis* displayed in vitro inhibitory activity against α-glucosidase, an indication of their potential for control of hyperglycemia [7]. Evidence from a study in mice demonstrated that a probiotic *E. faecium* strain shows in vivo antihyperglycemic effects and decreases insulin resistance when administered in conjunction with food [233]. An *E. faecalis* strain from traditional Tunisian Rigouta increased by 28% the secretion of interleukin-10 in Caco-2/TC_7_ cells, an indication of its immunomodulatory potential [235]. In a study by Graham et al. [7], milk fermented with *E. faecalis* strains from cheese exhibited a high total phenol content and radical scavenging activity.

Enterococci are among the several food-associated bacteria that can produce conjugated linoleic acid (CLA), a derivative of an essential fatty acid, linoleic acid, which has received considerable attention due to its potential health effects [236]. Kishino et al. [237] showed that enterococcal strains can produce high concentrations of CLA under laboratory conditions and Ross et al. [238] proposed use of CLA-producing LAB to manufacture novel, value-added cheeses.

The gut microbiome is now known to play an important role in the so-called gut-brain axis, and to impact their human host’s mental health by several mechanisms, including production of neurotransmitters [239]. *Enterococcus* spp. [240] are known to produce serotonin in the gut, thus participating in the cross talk between these two organs. Enterococci are also capable of producing γ-aminobutyric acid (GABA) [80,241,242], a neurotransmitter possessing multiple health benefits beyond its impact upon mental health. Other GABA-producing LAB have been isolated from traditional, artisanal cheeses [241,242,243], but GABA production has not yet been reported in enterococci isolated from cheese.

The multiple, putative health benefits associated with enterococci, coupled with their ability to survive passage through the proximal part of the GIT and to colonize the host’s gut, make this genus an interesting candidate for development of probiotic cultures. Because they harbor an underexploited, diverse microbiota [47], traditional, artisanal cheeses have been successfully screened for enterococcal strains with probiotic potential [205,235,243,244,245]. Enterococcal probiotics have, so far, met with limited commercial success, and only under the form of dietary supplements [246]; however, foods such as fresh cheese would also serve as suitable carrier for probiotic microorganisms [247,248]. The major constraints upon commercial application of enterococci in probiotic preparations and foods for human consumption arise from safety concerns, insufficient information on their safety-related properties and health benefits, and unfavorable regulatory environment [8]. Information on the safety significance of enterococcal traits (such as their adherence factors), concerns that they might act as reservoirs of virulence and antibiotic-resistance genes, their propensity to engage in horizontal gene transfer (both within their genus and with other bacteria), the need to further substantiate the health claims associated with potential probiotic candidates, and the lack of QPS/GRAS status make it very hard presently to successfully develop and place in the market an *Enterococcus*-based food product. The available data on safety and benefits of enterococci as probiotics are not sufficient to perform a thorough risk analysis. If enterococci, from cheese or other sources, are to be employed as probiotics, said knowledge gap must be bridged.

## 7. Enterococci as Opportunistic Pathogens

### 7.1. General Aspects

By the time that Thiercellin & Jouhaud [11] applied the denomination “enterocoque” to intestinal commensal cocci, the first case of a human infection attributable to this genus was also described; however, the emergence of enterococci as problematic, opportunistic pathogens would come only in the late 1970s [219]. Despite their increasing importance as nosocomial opportunists, enterococci are not regarded as highly virulent, as they require an immunocompromised host for disease to happen [219]. In this type of hosts, they cause primarily urinary tract infections, but also nosocomial bacteremia, and intra-abdominal, pelvic, wound, and tissue infections. Meningitis and respiratory infections caused by enterococci are very rare [249]. Rather than being linked to high virulence, the success of the enterococci as nosocomial pathogens can be explained by their evolutionary history. While co-evolving with their hosts to adapt to terrestrial life, the enterococci became a hardy genus. They acquired a propensity to exchange genetic information with other bacteria that occupy the same habitats, so they developed streamlined, malleable genomes that enabled them to thrive even when faced with such environmental stress factors as antibiotic exposure that characterize modern healthcare facilities [17]. 

Mundy et al. [250] indicated 12 enterococcal species implicated in human infections: *E. avium*, *E. casseliflavus*, *E. durans*, *E. faecalis*, *E. faecium*, *E. gallinarum*, *E. hirae*, *E. malodoratus*, *E. mundtii*, *E. pseudoavium*, *E. raffinosus*, and *E. solitarius*. Of these, *E. faecalis* and *E. faecium* are the most prevalent; they represent ca. 75% of the clinical isolates [249]. *E. faecalis* is one of the so-called ESKAPE pathogens (*E. faecium*, *S. aureus*, *Klebsiella pneumoniae*, *Acinetobacter baumannii*, *Pseudomonas aeruginosa*, and *Enterobacter* spp.), a group of virulent, multi-resistant microorganisms that account for most human nosocomial infections [251,252]. Infections by other enterococci, however, have been increasingly reported [219].

### 7.2. Virulence Factors of the Enterococci

Several virulence factors have been described in enterococci, mostly in *E. faecalis* and *E faecium* [247]. Chajęcka-Wierzchowska et al. [252] have classified the enterococcal virulence factors described so far in two groups: surface proteins that promote colonization of the host, and secreted metabolites that damage the host’s tissues. In extracellular pathogens such as enterococci, the preferential adherence targets are components of the extracellular matrix (ECM) or the serum, such as collagen, fibronectin, and fibrinogen [253]. Although adhesion to the host is the first stage of the infection process, it cannot be exclusively regarded as a virulent feature; as commensals, enterococci also need to adhere to the intestinal epithelium to avoid being carried away by peristaltic movements [252]. Once they have successfully colonized the host, virulent strains of enterococci secrete histotoxic metabolites, thereby damaging the host’s tissues. Virulence factors that promote adhesion (all of them surface proteins) include aggregation substance (AS), collagen-binding proteins (Accessory colonization factor, ACE in *E. faecalis* and ACm in *E. faecium*), cell wall adhesin (endocarditis specific antigen, Efa A), and enterococcal surface protein (Esp); whereas cytolysin (Cyl), gelatinase (gelE) and hyaluronidase (Hyl) are classified as part of the group of secreted toxins with negative effects upon the host’s tissues [252].

AS refers to a group of highly homologous adhesins, encoded in large plasmids, that mediate efficient adhesion between bacteria, and facilitate plasmid exchange as an integral part of the enterococcal pheromone system [253]. These proteins play, therefore, a role in disseminating plasmids that encode determinants of antibiotic resistance, as well as other virulence factors (e.g., cytolysin) within a bacterial species [254]. Besides mediating adhesion between conjugating bacterial cells, AS also promotes adhesion to various types of host cells, such as renal tubular cells and intestinal epithelial cells, as well as to extracellular matrix (ECM) proteins [254]. AS is thought to play a role in biofilm formation by enterococci [247]. Furthermore, it has a protective action against the host’s defense mechanisms. In *E. faecalis*, AS was demonstrated to promote opsonin-independent biding to human neutrophils, thus making the bacterium harder to kill by these defensive cells [255]. It can also promote binding to macrophages, thus increasing the survival time of *E. faecalis* inside these cells [255]. AS acts synergically with cytolysin, so AS^+^/Cyl^+^ strains exhibit increased mortality compared to isogenic strains that express only one of such genetic virulence determinants [245]. Genetic determinants for AS proteins have been found in *E. faecalis* and in *E. faecium* isolates; in clinical ones, they are more frequent in *E. faecalis* than in *E. faecium* strains [256,257,258]. Recently, the presence of *asa1* has been described in *E. gallinarum* strains of food origin [259].

ACE has been associated with *E. faecalis* and is present in most clinical isolates of this species [260]. It belongs to the Microbial Surface Component Recognizing Adhesive Matrix Molecules (MSCRAMM) group of surface proteins with LPXTG (lysine-proline-any amino acid-threonine-glycine) [253]. It is structurally more similar to the first MSCRAMM discovered in Gram-positive bacteria—the Cna protein of *S. aureus*—than to the ACm protein of *E. faecium* [252]. It promotes colonization of the host by binding to ECM and participates in binding to type I and IV collagen and to laminin [253]. It is encoded in the *ace* gene; deletion of *ace* decreased virulence, and affected survival of *E. faecalis* inside the host’s macrophages. This virulence determinant has been demonstrated to be involved in urinary tract infections [253] and in endocarditis [260]. *E. faecium* also possesses a collagen-binding protein (ACm). Unlike ACE, it only binds to collagen, not to lamin, and shows greater affinity towards collagen I than collagen IV [260].

EfaA is a potential virulence determinant, thought be involved in pathogen’s adherence to the host’s cells; however, knowledge of its role in enterococcal infections is still scarce [261]. It has been suggested that this protein plays a role in adhesion in endocarditis [262]. EfaA is encoded by *efaA_fs_* in *E. faecalis* strains and *efaA_fm_* in *E. faecium* strains [263].

Esp is the largest of the enterococcal proteins identified. Its structure is similar to that of several proteins of other Gram-positive bacteria, such as C-α in β-hemolytic *Streptococcus agalactiae*, R28 in *Streptococcus pyogenes*, and Bap in *S. aureus*. The latter is associated with biofilm formation [252], and its role therein has been duly demonstrated [264]. In biofilms, bacteria display higher resistance to antibiotics than in their planktonic form, and exchange of genetic material between bacterial cells is favored [265]. The genetic determinant of Esp (*esp*) is in a pathogenicity island, which also contains proteins responsible for antibiotic efflux [266]. The relation between carriage of *esp*, vancomycin and multiple antibiotic resistance has been demonstrated in *E. faecium* strains [267]. The mechanisms for transferring the *esp* differ between *E. faecalis* strains (chromosome-to-chromosome transmission) and *E. faecium* (integration into a conjugative plasmid) [268].

Cyl is one of the best characterized enterococcal virulence factors. It is a bacteriocin-type exotoxin, with bactericidal activity against Gram-negative bacteria and hemolytic action against erythrocytes (β-hemolysis), leukocytes and macrophages. The operon that encodes for Cyl is in mobile genetic elements, either in strongly conserved, pheromone-dependent plasmids (such as pAD1), or within the same chromosomal pathogenicity island that contains the determinants for such other virulence factors as Esp or AS. It is widely spread and can be found in many enterococcal species (*E. faecalis*, *E. faecium*, *E. avium*, *E. casseliflavus*, *E. cecorum*, *E. durans*, *E. gallinarum*, *E. malodoratus*, and *E. raffinosus*) from clinical, animal and food origin [252,269].

The extracellular, zinc-dependent metalloendopeptidase Gel is encoded by *gelE*, which is in the enterococcal chromosome. It acts on elatine, elastin, collagen, and hemoglobin, and may play a role toward biofilm formation [252]. It occurs mostly in clinical and food strains of *E. faecalis*, but is only rarely found in *E. faecium* [263].

Hyl targets the mucopolysaccharides in the connective tissue and cartilage, thus promoting dissemination of enterococci within the host’s tissues [252]. In clinical isolates, it occurs chiefly in *E. faecium*, and very seldom in certain *E. faecalis* strains [270]. It has also been found in strains of other species (*E. casseliflavus*, *E. mundtii* and *E. durans*) isolated from foods [252].

### 7.3. Virulence Determinants in Enterococci from Foods

Due to their importance as human opportunistic pathogens, more studies are available on the presence of genetic determinants of virulence in *E. faecalis* and *E. faecium* than on other enterococcal species. In most such studies, *E. faecalis* strains were found to carry a much higher number of genetic virulence determinants than *E. faecium* strains, both in clinical [258,264,271,272] and food strains [261,263,264,272], including those from traditional cheeses [102,271,273,274]. In many studies, genes that encode for adhesion factors (e.g., *asa1*/*agg*, *esp* and *efaA*) were highly prevalent among *E. faecalis* isolates from cheeses [152,154,166,274,275,276,277,278] but could also be found in *E. faecium* isolates [102,203,276,278,279,280]. Much fewer studies exist on the prevalence of these genetic determinants in other enterococcal species isolated from cheeses. EfaA determinants (*efaAfm* and/or *efaAfs*) were found in *E. casseliflavus* [276], *E. durans* [275,276,280], *E. gallinarum* [275], *E. hirae*, and *E. italicus* [280]. AS determinants (*agg/asa1*) were found in *E. durans*, *E. italicus* [280], and *E. avium* [142]. The *esp* gene was found only in *E. durans* [280]. None of the non-*faecalis*, non-*faecium* isolates in these studies carried *ace*. In some studies, none of the *E. casseliflavus*, *E. durans*, *E. gallinarum* [168,273], and *E. italicus* [193,281] strains tested carried genetic determinants of adhesion. 

In *E. faecalis* and *E. faecium* isolates from cheese, *gelE* has been frequently found [102,142,152,166,203,235,274,275,276,277,280,282]. The reported prevalence of Cyl determinants was lower than that of *gelE* in these two species [142,152,203], and presence of *hyl* was even rarer [203]. Studies on the carriage of genetic determinants for Cyl, Gel and Hyl by non-*faecium*, non-*faecalis* strains are scarce. The *gelE* gene has been described in *E. casseliflavus* [276], *E. durans* [275,280], *E. hirae* [142], and in one *E. italicus* strain [280]. Even less frequently reported was the existence of Cyl and Hyl determinants. Nieto-Arribas et al. [142] described presence of *cylA* in two isolates belonging to the *E. avium* and *E. hirae* species, and *hyl* in two *E. avium* strains.

The existing data on prevalence of the aforementioned virulence factors in enterococcal strains from cheese should be interpreted with care—especially in the case of non-*faecalis*, non-*faecium* isolates—since the number of studies available is low. Nevertheless, they suggest that in cheese enterococci, genetic determinants of adhesion proteins are common, whereas genetic determinants of histo- and cytotoxic metabolites, except for *gelE*, seem much less disseminated. Presence of adhesion determinants is not frequently regarded as an indication of pathogenic potential *per se*, but simply as an adaptation to life as commensal, by contributing to persistence of enterococci in the host’s GIT [23,102,277,283,284]. Furthermore, no enterococcal infections have been traced to food sources [252], leading to a widespread perception that the enterococci from artisanal cheeses pose a very low risk to public health. Bearing in mind that sex pheromone determinants (*cpb*, *cob*, *ccf*) have been found in enterococci isolated from cheeses often with a high prevalence [23,166,276,278,279], and that the genes encoding virulence factors are frequently located in pathogenicity islands or mobile elements that facilitate their dissemination even further [219], the possibility that cheeses may serve as a route for the transfer of virulence genes from their enterococcal reservoirs to the bacteria in the GIT of their consumers should not be discarded [153,193,252,285,286]. Further studies are needed not only to clarify the potential role of cheeses and their enterococci toward dissemination of virulence genes, but also to support strategies aimed at mitigating this problem. It is noteworthy that in two studies on traditional cheeses, a decrease in number of *Enterococcus* isolates harboring virulence determinants was observed towards the end of their 60–120 d-period of maturation [102,276]; this raises the question of whether other manufacture parameters could also influence the potential role of artisanal cheeses as reservoir of bacterial virulence genes within their enterococcal populations.

Because presence of virulence factors in enterococci is strain-dependent, a genus- or species-wide decision on their safety status has been hampered. The Scientific Committee of the European Food Safety Agency has accordingly decided not to grant them Qualified Presumption of Safety (QPS) [8], and neither has the US Food and Drug Administration granted them Generally Regarded as Safe (GRAS) status [8,235]. The lack of QPS/GRAS status significantly hinders application of enterococci as food cultures and conveys one more justification to why research on enterococcal safety-related traits is of utmost importance.

## 8. Enterococci as Reservoirs of Antibiotic Resistance

**Figure 2:**
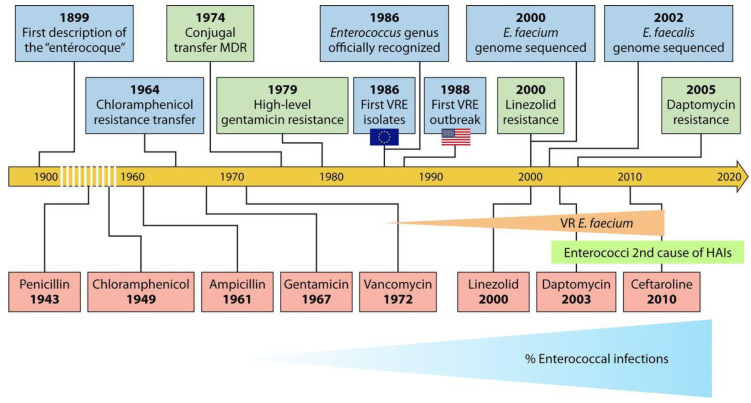
History of enterococci as human pathogens, with emphasis on emergence of antibiotic resistance in the genus. HAIs: hospital acquired infections. Reprinted with permission from García-Solache et al. [219]. (License No. 5016471445387).

The evolutionary history of enterococci has primed them to colonize modern hospital environments, and cause infection in immunocompromised patients by endowing these bacteria with a notable resistance to environmental factors; this set of genetic determinants enables them to colonize efficiently their hosts, and a striking ability to trade genes with other bacteria, thus fostering their adaptation to the seemingly hostile conditions faced therein [219]. The ability to resist antibiotics, acquired during evolution of this genus, is one of the factors that explain the success of enterococci as nosocomial pathogens. Antibiotic resistance in enterococci was acknowledged even before they were recognized as a genus, and the first report on what is thought to have been an enterococcal infection was almost simultaneous with the coining of this bacterial epithet by Thiercelin and Jouhaud [282]. Because enterococci were no longer killed by many of the β-lactam antibiotics that are effective against other Gram-positive cocci, treatment of infections with these agents resorted to synergistic combinations of β-lactam antibiotics with aminoglycosides [219]. The emergence of high-level aminoglycoside resistance, a few years later, rendered this combination therapy ineffective, and promoted an antibiotic that had found little use so far—vancomycin—to the role of leading therapeutic agent against enterococcal infections by the late 1970s–early 1980s. The first reports on vancomycin-resistant enterococci (VRE) appeared in the 1980s, and every new antibiotic introduced afterward to replace vancomycin as a last-line therapy has been followed by reports of resistant enterococcal strains (Figure 2), thus rendering it ineffective for this purpose. At present, multidrug-resistant enterococci are a leading cause of nosocomial infections, thus raising challenges for their treatment and constituting a heavy social burden [282]. 

Early on, *E. faecalis* was the causative agent of most enterococcal infections. However, *E. faecium* has emerged during the last two decades as leading agent of multidrug-resistant enterococcal infections [286]. One possible explanation for this shift lies on the emergence of VRE. From the earliest reports, most clinical VRE strains belonged to the *E. faecium* species. The success of VR *E. faecium* in penetrating and colonizing hospitals has led to a situation that can presently be described as endemic. Factors that led to this endemicity in hospitals have been identified as clonal dissemination of particular strains of said pathogen, selective pressure exerted by prolonged antibiotic regimens, and limitations of usual infection control strategies against this unusually hardy pathogen [250]. In a comparative genomics study by Lebreton et al. [4], it was demonstrated that the currently circulating multidrug resistance (MDR) strains that infect humans belong to a clade that probably diverged from *E. faecium* strains that adapted to other animal hosts ca. 80 years ago, at a time when antibiotics were introduced as therapeutic agents. The genomes of this clade are highly malleable, thanks to an increase in mobile genetic elements, to hypermutability, and to metabolic alterations [287].

Bacterial resistance to antibiotics is usually classified as either intrinsic or acquired, with the former resistance being encoded by chromosomal genes present in all members of a species. This resistance is likely due to selective pressures that all members of an enterococcal species experience when colonizing the gut, namely their need to survive innate defenses of the host (consubstantiated in the action of lysozyme, phospholipase C, antimicrobial peptides, or bile-like detergents). It may also result from exposure to selective pressures outside the host, such as resistance to starvation and desiccation [17]. Conversely, acquired resistance is encoded either by genes located in such mobile elements such as plasmids and transposons, or is a result of mutations. The most common transmissible elements implicated in exchange of genetic determinants of resistance in enterococci are the *Tn*3 family transposons, such as *Tn*917 (resistance to macrolides-lincosamide-streptogramin B, MLSB antibiotics), *Tn*1546 (resistance to glycopeptide resistance), and conjugative transposon *Tn*916 (minocycline and tetracycline) [285]. Plasmids are abundant in enterococci and entail another important contribution to their genomic plasticity. Transmission of antibiotic-resistance determinants has been mediated chiefly by pheromone-responsive plasmids and broad host range plasmids of the incompatibility group 18 type (Inc. 18 type). Pheromone-responsive plasmids are found mainly in *E. faecalis* and are highly efficient in transmitting genetic information within this species. Broad host range plasmids can mediate transfer of genetic information between bacteria, but not so efficiently as pheromone-responsive plasmids. Broad host range plasmids of the Inc. 18 type have been implicated in transfer of vancomycin resistance determinants from enterococci to methicillin-resistant *S. aureus* [288]. Oftentimes, clinical isolates contain multiple plasmids and transposons [289].

Bacteria can acquire genetic determinants of antibiotic resistance via spontaneous mutations, yet development of multidrug resistance (MDR) by this mechanism only would require a long time. By contrast, horizontal gene transfer (HGT) allows bacteria exchange antibiotic-resistance genes in a more time-efficient manner, by promoting collaboration of the whole bacterial community toward development of MDR [290]. Bacteria can gain mobile elements by conjugation or transduction; such genetic transfer is more likely to occur in environments where bacteria are present to large numbers and undergo selective pressure [291]. Although this process is not yet well-studied, the gut microbiota probably becomes enriched in intrinsically resistant enterococci and other overtly resistant species (many of which carry mobile elements) under antibiotic selective pressure, and this contributes to the ability of enterococci serve as “trade hubs” for a variety of antibiotic-resistance plasmids and transposons [282].

Enterococci developed effective mechanisms for HGT, and this explains (at least in part) the increasing dissemination of antibiotic-resistance genes within this genus [281]. Conjugation is the main HGT mechanism in enterococci. Natural transformation as a means of acquiring mobile genetic elements by enterococci has not yet been described, nor is the role of transduction clear yet [292].

**Figure 3:**
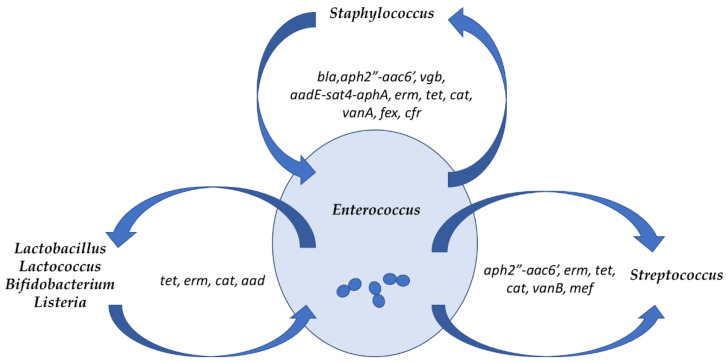
Shared genetic determinants of antibiotic resistance between enterococci and other bacteria that occupy the same habitats, with indication of the main genetic determinants of resistance shared; *tet* refers to either *tetK*, *L*, *M, O*, and/or *W*, and *erm* refers to *ermA, B* and/or *C.* (Data for this figure were obtained from Werner et al. [5]).

The genomic plasticity of the enterococcal genome, both in clinical and food isolates, is due in part to lack of a functional CRISPR system [293,294], complemented by an efficient plasmid transfer system that involves production of pheromones. As a result, they have a great capacity to acquire and spread genetic traits, including resistance genes [295]. Enterococci have indeed been found to trade genetic determinants both in vitro and in vivo, not only within their own genus, but also with bacteria belonging to other genera with whom they share habitats, viz. other LAB (lactobacilli, lactococci), streptococci, staphylococci, *Listeria*, and bifidobacteria [5] (Figure 3).

*Enterococcus* species are intrinsically resistant to cephalosporins, low levels of most other β-lactams, low levels of aminoglycosides, sulfonamides, clindamycin, quinupristin, and dalfopristin. In addition, *E. casseliflavus* and *E. gallinarum* possess intrinsic resistance to low levels of vancomycin [289]. Intrinsic resistance of enterococci to β-lactams (including cephalosporins) is due to expression of low-affinity penicillin-binding proteins (PBPs) that bind weakly to such antibiotics. Since enterococci do not synthesize folate, they are constitutively resistant to sulfonamides. Intrinsic resistance to lincosamides, streptogramin A, and to the combination quinupristin-dalfopristin in *E. faecalis* is probably due to drug efflux, encoded in a chromosomally located gene (*lsa*). In *E. faecalis*, resistance to clinically achievable concentrations of aminoglycosides is intrinsic, and has been attributed to the low permeability of its cell wall to this type of molecules. Such a low-level resistance can be overcome by combining an aminoglycoside with a penicillin [296]. Resistances meanwhile acquired by *Enterococcus* include resistance to chloramphenicol, erythromycin, tetracycline, fluoroquinolones, glycopeptides and high levels of clindamycin, aminoglycosides, and β-lactams [219,289].

Enterococci can increase their resistance to penicillins by acquiring the plasmid-borne *bla* genes that encode β-lactamases [289], or by mutations in PBP4 (*E. faecalis*) and PB5 (*E. faecium*) [219]. Production of β-lactamases is rare in enterococci and occurs mostly in *E. faecalis* [289].

The development of resistance to high levels of aminoglycosides in *E. faecalis* and *E. faecium* typically results from acquisition of mobile genetic elements encoding for antibiotic-modifying enzymes. The major determinant of high-level resistance to this class of antibiotics is the bifunctional gene *aph(2″)-Ia-aac(6’)-Ie*; it encodes for an antibiotic-modifying enzyme conferring resistance to all clinically relevant aminoglycosides, except streptomycin [289]. The enzyme involved in high-level resistance to streptomycin is usually encoded in the *ant-6* gene [219].

From a medical perspective, dissemination of acquired resistance to glycopeptides, such as vancomycin and teicoplanin (by vancomycin-resistant enterococci, VRE) has been one of the most alarming developments, because of the importance of these bactericidal drugs when aminoglycoside/β-lactam therapy is compromised by high-level aminoglycoside resistance. The target of vancomycin is the cell wall, where it interferes with establishment of the peptide crosslink, thus affecting stability of this bacterial structure. Nine operons conferring vancomycin resistance to enterococcal strains have been described so far. Four of them—*vanA*, *vanB*, *vanC* and *vanM*—encode replacement of the terminal D-alanine in the pentapeptide precursors with D-lactate, and confer resistance to both vancomycin and teicoplanin. The remaining operons (*vanC*, *vanE*, *vanG*, *vanL*, and *vanN*) lead to precursors containing a terminal D-serine. The strains that express said determinants display low-level resistance to vancomycin, but not to teicoplanin [219,289,295]. VRE genotypes *vanA* and *vanB* are the most prevalent in Europe, and VanA-type *E. faecium* is the most problematic for humans. Unlike what happens with *E. faecium*, both ampicillin resistance and vancomycin resistance are seldom found in *E. faecalis* [5].

Despite the profusion of studies on antibiotic resistance and its genetic determinants of clinical enterococci, few studies are available that focus on enterococcal species carried by traditional cheeses [44,152,166,179,193,273,297]. As shown in Table 2, *E. faecalis* isolates were proven to carry tetracycline-resistance determinants [44,179,193,273]. Both efflux protein (*tetK*, *tetL*) and ribosomal determinants (*tetM, tetS*) were found in these studies [44,130,193,273]. The genetic determinant for chloramphenicol resistant *cat* (which encodes for a chloramphenicol acetyltransferase) was also present in *E. faecalis* isolates from traditional cheeses [44,273]. Other genetic determinants detected were *aadE* (encoding for aminoglycoside 6-adenyltransferase involved in streptomycin resistance) [273], *aph3′* (an aminoglycoside phosphotransferase), and *ermB* (which encodes for a ribosomal protection protein involved in resistance to erythromycin) [44]. Vancomycin resistance determinants (*vanA*) were reported only in a single study [142], and just in two strains. *E. faecium* carrying genetic determinants of antibiotic resistance were rare [179,273], with detection of *tetM* in only one strain [273]. In *E. italicus*, only the *tetS* (which encodes for a ribosomal protection protein) and *tetK* tetracycline-resistance determinants were found [193,297]. No genetic determinants of resistance were recorded for the *E. gilvus* strains studied [179].

Phenotypic resistance to aminoglycosides (low level), cephalosporins, penicillins, tetracyclines, trimethoprim, and sulfamethoxazole-trimethoprim was detected in *E. faecalis* isolates from cheese (Table 2). Resistance to these antibiotics is considered intrinsic in enterococci [289]. On the other hand, resistance to chloramphenicol, erythromycin, glycopeptides, quinupristin-dalfopristin, and tetracyclines is acquired. Among these, chloramphenicol and vancomycin are part of the World Health Organization (WHO) Model List of Essential Medicines [299], so preventing dissemination of resistance to these chemotherapeutic agents is important. Detection of phenotypic resistance to chloramphenicol and vancomycin is in line with presence of the corresponding genetic determinants shown in Table 2; and may raise concerns on the possibility of dissemination of resistance to these important antibiotics, even though resistance to vancomycin was infrequent [152]. Resistance to tetracycline also agrees with the wide dispersion of its genetic determinants among *E. faecalis* from traditional cheeses (Table 2); presence of multi-resistant strains (i.e., resistant to more than 3 antibiotics) in some studies is also noteworthy [44,152,179,298].

Regarding *E. durans* and *E. faecium* isolates, resistance to penicillin, erythromycin, tetracycline, levofloxacin, norfloxacin, ciprofloxacin, chloramphenicol, and nitrofurantoin was reported (Table 2). Resistance to the latter three is of concern, for not being intrinsic in enterococci, and they are regarded as essential antibiotics for humankind [299,300]. Despite the presence of *tetM*, tetracycline resistance was not reported in *E. faecium* as per these studies. Resistance against tetracycline in *E. italicus* was, however, consistent with presence of genetic determinants for drug efflux and ribosomal protection. Furthermore, all *E. italicus* strains were resistant to rifampicin and trimethoprim in one of the studies summarized in Table 2, so the authors claimed that this could be a characteristic trait of this species [193].

The number of available studies that analyzed in depth the antibiotic resistance and the enterococcal resistome in artisanal cheeses is low. Therefore, existing data cannot backup a thorough analysis of the risks associated with presence of antibiotic-resistant strains and transmissible genetic determinants of resistance.

However, presence of transmissible genetic determinants of resistance to clinically relevant antibiotics, such as aminoglycosides and vancomycin; the range of phenotypic resistance in *E. faecalis* strains; and presence of multi-resistant enterococci all raise concerns. Research on this issue is urgent, not only to help manage the spread of antibiotic resistance, but also to better document the safety status of strains that, due to their technological properties and probiotic potential, could provide a tool for quality improvement and innovation. Although these strains are part of the microbiota of cheeses that have a century-long history of safe consumption, the circumstances have changed in the latest 80 years, since discovery of antibiotics and their introduction into clinical practice has raised a new selective pressure that is likely to shape enterococcal evolution in ways that may eventually impact human health.

## 9. Conclusions

Enterococci are an important part of the microbiota in many traditional cheeses, and their metabolic activities contribute to the unique sensorial properties associated with each type of cheese. Since they do not behave as strong acidifiers, they are not an ideal choice for starters, yet they may offer a promise toward development of cheese adjunct/protective cultures. Enterococci in cheese can impact the health of its consumers in many favorable ways, thus making them an interesting source of (potentially) probiotic strains. Enterococci are not highly virulent; and there are no reports of enterococcal infections linked to cheese consumption. Nevertheless, cheese enterococcal strains may harbor a few genetic determinants of virulence and antibiotic resistance, often located in mobile genetic elements. Enterococci have been touted as “gene traffickers”, and can accordingly obtain and disseminate virulence and antibiotic virulence genes in the GIT of their hosts and in the environment. In this sense, they have the potential to constitute a health threat that urges some form of mitigation. However, knowledge of enterococci in artisanal cheeses is relatively scarce. By making use of the increasingly accessible high-throughput, culture-independent technologies in conjunction with classical bacteriology techniques, one should further investigate enterococcal diversity and population dynamics in these cheeses. A better understanding of their influence upon human health is a must, not only to minimize the risks associated with their ability to disseminate virulence and antibiotic-resistance genes, but also to permit a better exploitation of their potential as adjunct, protective, and probiotic cultures.

## Figures and Tables

**Table 1:** Enterococcal species identified in European artisanal/traditional cheese varieties.

Cheese (Type *)	Country	Milk Species/Treatment	Maturation Time	Enterococcal Species	Ref.
Alberquilla (H)	Spain	Ewe/goat	-	*E. devriesei, E. faecium*	[117]
Arzúa-Ulloa (S)	Spain	Cow (raw, pasteurized)	6 mo	*E. durans*	[122]
Bitto (H)	Italy	Cow (raw)	>70 d	*E. durans, E. faecalis, E. faecium, E. lactis*	[73,123]
Blue-veined cheese (MR)	UK	Cow (raw)	3 mo	*E. durans, E. faecalis*	[120]
Bryndza (S)	Slovakia	Ewe (raw)	≤14 d	*E. casseliflavus, E. durans, E. faecalis, E. faecium, E. mundtii, E. pallens*	[124,125,126]
Cabrales (MR)	Spain	Cow/ewe/goat (raw)	2–5 mo	*E. durans, E. faecalis*	[122]
Caciocavallo Palermit. (PF)	Italy	Cow (raw)	4 mo	*E. casseliflavus, E durans, E. faecalis, E. gallinarum*	[75,127]
Calenzana (S)	Italy	Ewe/goat	4–8 mo	*E. faecium, E. durans, E. hirae*	[76]
Canestrato Pugliese (H)	Italy	Ewe (raw)	4 mo	*E. faecalis, E. faecium*	[128]
Casera Valtellina (H)	Italy	Cow (raw)	70 d	*E. faecalis, E. faecium, E. gilvus*	[129,130]
Casín (SH/H)	Spain	Cow (raw)	38 d	*E. faecium*	[91]
Cazisolu (PF)	Italy	Cow (raw)	1 mo	*E. durans, E. faecium, E. italicus, E. lactis*	[131]
Cueva de la Magahá (H)	Spain	Goat (raw)	8 mo	*E. devriesei, E. faecalis, E. faecium, E. hirae, E. malodoratus*	[92]
Danbo (SS)	Denmark	Cow	18 wk	*E. faecalis, E. faecium*	[132]
Feta (S; B)	Greece	Ewe/goat	2 mo	*E. canis, E. faecalis, E. faecium*	[133]
Fiore Sardo (H)	Italy	Ewe (raw)	9 mo	*E. durans, E. faecalis, E. faecium*	[77]
Fontina (SS)	Italy	Cow (raw)	≤9 mo	*E. durans, E. faecalis, E. faecium, E. hirae*	[48,134,135]
Fossa (H)	Italy	Cow/ewe	1–3 mo	*E. durans, E. faecium, E. faecalis*	[136]
Fresh cheese (F)	Croatia	Cow (pasteurized)	none	*E. durans, E. faecium, E. hirae, E. ratti, E. villorum*	[137]
Galotyri (F)	Greece	Ewe/goat	none	*E. durans, E. faecalis, E. faecium*	[98]
Genestoso (S)	Spain	Cow (raw)	20–30 d	*E. faecalis*	[138]
Gorgonzolla (MR)	Italy	Cow	3–4 mo	*E. faecalis, E. faecium*	[129]
Gouda-type (SH/H)	Belgium	Cow (raw)	2–4 mo	*E. casseliflavus/malodoratus* group, *E. faecalis, E faecium/durans* group	[121,139]
Graviera (H)	Greece	Ewe/goat (thermized)	3 mo	*E. durans, E. faecalis, E. faecium, E. hirae*	[99,100]
Hervé (S)	Belgium	Cow (raw)	63 d	*E. casseliflavus, E. faecalis, E. faecium*	[140]
Idiazábal-type (S; Smk)	Spain	Ewe (raw)	70–72 d	*E. durans, E. faecalis, E. faecium, E. casseliflavus, E. hirae, E. gallinarum*	[37]
Istrian (H)	Croatia	Ewe (raw)	3 mo	*E. faecalis, E. faecium, E. italicus, E. saccharominimus, E. sulfureus*	[141]
Kalathaki Lemnou (S)	Greece	Ewe/goat (raw)	3 mo	*E. durans, E. faecalis, E. faecium, E. gilvus*	[101]
Livanjski (SH)	Bos.-Herz.	Cow/ewe (raw)	1–2 mo	*E. durans, E. faecalis, E. faecium, E. gilvus, E. hirae, E. lactis, E. malodoratus*	[95]
Manchego (SH/H)	Spain	Ewe (raw, pasteurized)	2–24 mo	*E. avium, E. faecalis, E. faecium, E. hirae*	[142]
Maroilles (S)	France	Cow (raw)	≤4 mo	*E. devriesei*	[143]
Melichloro (H)	Greece	Ewe/goat (raw)	4–6 d	*E. avium, E. durans, E. faecium, E. pseudoavium*	[101]
Montasio (SH)	Italy	Cow (raw)	2–5 mo	*E. durans, E. faecalis, E. faecium, E. gallinarum*	[78,79]
Mozzarella (F; PF)	Italy	Cow (raw)	fresh	*E. faecalis, E. sulfureus*	[144]
Nostrano (S)	Italy	Cow (raw)	8 mo	*E. faecalis*	[80]
Nostrano di Primiero (S)	Italy	Cow (raw)	2 mo	*E. faecalis, E. faecium*	[81]
Oscypek (SH; Smk)	Poland	Ewe (raw)	5–18 d	*E. durans, E. italicus*	[145]
Pecorino (central It.) (H)	Italy	Ewe (raw)	1–12 mo	*E. durans, E. faecalis, E. faecium*	[146]
Pecorino Abruzzese (H)	Italy	Ewe (raw)	3 mo	*E. durans, E. faecalis, E. faecium*	[102]
Pecorino Crotonese (H)	Italy	Ewe (r., therm., past.)	4 mo	*E. faecalis*	[147]
Pecorino di Tramonti (H)	Italy	Ewe (raw)	1–3 mo	*E. durans, E. faecalis, E. faecium*	[105]
Pecorino Siciliano (H)	Italy	Ewe (raw)	0.5–6 mo	*E. durans, E. faecalis, E. faecium, E. hirae*	[82,106,148]
Picante (SH)	Portugal	Ewe/goat (raw)	4–6 mo	*E. durans, E. faecalis, E. faecium,*	[149,150,151]
Pico (SS)	Portugal	Cow (raw)	21 d	*E. faecalis, E. italicus, E. pseudoavium*	[152,153,154]
Provolone del Monaco (SH)	Italy	Cow (raw)	6–12 mo	*E. durans, E. faecalis, E. faecium*	[83]
Quesailla Arochena (NS)	Spain	Goat (raw)	4 mo	*E. devriesei, E. faecalis, E. malodoratus*	[118]
Ragusano (H; PF)	Italy	Ewe (raw)	6 mo	*E. durans, E. faecalis, E. faecium, E. hirae*	[148]
Raschera (H)	Italy	Cow (raw)	1–6 mo	*E. casseliflavus, E. faecalis, E. faecium*	[84]
Raw goat milk (SH)	Spain	Goat (raw)	2 mo	*E. casseliflavus, E. durans, E. faecalis, E. faecium, E. gallinarum, E. hirae, E. italicus, E. lactis*	[93]
S. Jorge (SH/H)	Portugal	Cow (raw)	6–12 mo	*E. faecalis, E. faecium*	[155]
Saint-Nectaire (S)	France	Cow (raw)	28 d	*E. faecalis*	[156]
Salers (SH)	France	Cow (raw)	5 mo	*E. faecalis, E. faecium*	[157,158,159]
San Simón da Costa (SH;Smk)	Spain	Cow (raw)	45–60 d	*E. faecalis, E. sulfureus*	[160]
Scimudin (S)	Italy	Cow (raw)	>10 d	*E. faecalis, E. faecium*	[129]
Semicotto caprine (H)	Italy	Goat (raw)	2 mo	*E. durans, E. faecalis, E. faecium, E. gallinarum, E. hirae*	[85]
Serbian artisanal (NS)	Serbia	Cow (raw)	-	*E. durans, E. faecalis*	[161]
Serpa (SS)	Portugal	Ewe (raw)	30 d	*E. faecalis, E. faecium, E. hirae*	[111]
Serra (S)	Portugal	Ewe (raw)	2–6 mo	*E. faecium*	[162,163]
Sokobanja (NS)	Serbia	Cow (raw)	3d	*E. faecalis, E. faecium*	[116]
Stilton (MR)	UK	Cow (pasteurized)	9–12 wk	*E. faecalis*	[164]
Taleggio (SS)	Italy	Cow (raw, past.)	6–10 wk	*E. faecalis*	[129]
Terrincho (SS)	Portugal	Ewe (raw)	1 mo	*E. casseliflavus, E. durans, E. faecalis, E. faecium, E. gallinarum*	[165]
Tetilla (S)	Spain	Cow (raw)	1 mo	*E. durans, E. faecalis*	[122]
Tolminc (H)	Slovenia	Cow (raw)	2 mo	*E. faecalis*	[166]
Toma Piemontese (SS)	Italy	Cow (pasteurized)	20–45 d	*E. durans, E. faecalis, E. faecium*	[167]
Torta Arochena (NS)	Spain	Goat (raw)	4 mo	*E. avium, E. devriesei, E. faecalis, E. malodoratus*	[118]
Valsesia (SH)	Italy	Goat (raw)	1–2 mo	*E. casseliflavus, E. durans, E. faecalis, E. faecium, E. gallinarum, E. gilvus*	[86]
Vlasina (S; B)	Serbia	Goat (raw)	2 mo	*E. durans, E. faecalis, E. faecium*	[168]
White pickled (S; B)	Serbia, Croatia	Cow (raw)	1–10 d	*E. durans, E. faecalis, E. faecium*	[169]
Zlatar (SH; B)	Serbia	Cow (raw)	2 mo	*E. faecalis, E. faecium*	[170]

* H—hard; S—soft; SH—semihard; SS—semisoft; B—brined; F—fresh; MR—mold ripened; PF—pasta filata; Smk—smoked; NS—not specified.

**Table 2:** Antibiotic-resistance determinants and phenotypes reported for enterococcal strains from traditional cheeses.

Enterococcal Species	Genetic Determinants of Resistance	Phenotypic Resistance to ^1^	Reference(s)
*E. durans*	Not tested	ERY; CIP; PEN; TET; CHL	[102]
*E. faecalis*	*tetK,L,M,S*; *aadE*; *aph3′*; *cat; erm*; *vanA*	GENT; STR; KAN; NEO; CFT; CFO; PEN; OXA; TET; MYN; ERY; VAN; CHL; Q-D; SxT	[44,102,152,166,179,273]
*E. faecium*	*tetM*	ERY; CIP; LEV; NOR; NIT	[179,273,298]
*E. gilvus*	None detected	None detected	[179]
*E. italicus*	*tetK,S*	RIF; TET; TRI	[193,297]

^1^ Aminoglycosides (GEN—gentamycin, STR—streptomycin, KAN—kanamycin, NEO—neomycin); cephalosporins (CFT—ceftriaxone, CFO—cefotaxime); penicillins (PEN—penicillin G, OXA—oxacillin); tetracyclines (TET—tetracycline, MYN—minocycline); fluoroquinolones (CIP—ciprofloxacin, LEV—levofloxacin, NOR—norfloxacin); CHL—chloramphenicol; ERY—erythromycin; NIT—nitrofurantoin; RIF—rifampicin; SxT—sulfamethoxazole-trimethoprim; TRI—trimethoprim; VAN—vancomycin.
